# Monomer Conversion, Dimensional Stability, Biaxial Flexural Strength, Ion Release, and Cytotoxicity of Resin-Modified Glass Ionomer Cements Containing Methacrylate-Functionalized Polyacids and Spherical Pre-Reacted Glass Fillers

**DOI:** 10.3390/polym13162742

**Published:** 2021-08-16

**Authors:** Wisitsin Potiprapanpong, Whithipa Thepveera, Chutikarn Khamsuk, Somruethai Channasanon, Siriporn Tanodekaew, Somying Patntirapong, Naruporn Monmaturapoj, Piyaphong Panpisut

**Affiliations:** 1 Faculty of Dentistry, Thammasat University, Pathum Thani 12120, Thailand; wisitsin.pot@dome.tu.ac.th (W.P.); whithipa.the@dome.tu.ac.th (W.T.); p_somying@hotmail.com (S.P.); 2 Assistive Technology and Medical Devices Research Center (A-MED), National Science and Technology Development Agency, Pathum Thani 12120, Thailand; chutikarn.kha@ncr.nstda.or.th (C.K.); naruporn.mon@nstda.or.th (N.M.); 3 National Metal and Materials Technology Center (MTEC), National Science and Technology Development Agency, Pathum Thani 12120, Thailand; somruec@mtec.or.th (S.C.); siriporn@mtec.or.th (S.T.); 4 Thammasat University Research Unit in Dental and Bone Substitute Biomaterials, Thammasat University, Pathum Thani 12120, Thailand

**Keywords:** resin-modified glass ionomer cements, 2-hydroxyethylmethacryalte, monomer conversion, biaxial flexural strength, mass and volume changes, cytotoxicity, pulp protection

## Abstract

The aim of this study was to prepare RMGICs for pulp protection that contain polyacids functionalized with methacrylate groups (CMs) to enable light-activated polymerization without the need for toxic 2-hydroxyethyl methacrylate (HEMA) monomers. The effects of using CM liquids with 0 or 5 wt% HEMA on the physical/mechanical properties and cytotoxicity of the experimental RMGICs were assessed. Spherical pre-reacted glass fillers (SPG) were used as the powder phase. The experimental RMGICs were prepared by mixing SPG with CM liquid (0 wt% HEMA, F1) or CMH liquid (5 wt% HEMA, F2). Commercial materials (Vitrebond, VB; TheraCal LC, TC) were used for the comparisons. The degree of monomer conversion and fluoride release of both F1 and F2 were significantly lower than those of VB. F1 showed comparable biaxial flexural strength with VB but higher strength than TC. The dimensional stability (mass/volume changes) of the experimental materials was comparable with that of the commercial materials. F1 and F2 exhibited higher Sr/Ca ion release and relative cell viability than VB. The use of CMH liquid reduced the strength but enhanced the fluoride release of the experimental RMGICs. In conclusion, the experimental RMGICs showed comparable strength but lower cytotoxicity compared to the commercial RMGICs. These novel materials could be used as alternative materials for pulp protection.

## 1. Introduction

Untreated dental caries remain a major oral health problem globally [1]. The continuous mineral loss from the process of caries causes the severe destruction of the tooth structure, forming deep uncleanable carious cavities that may require restorative treatment. The current restorative management of deep caries lesions uses minimally invasive protocols such as selective caries removal techniques. The technique involves leaving demineralized dentin in the deepest area to reduce the risk of iatrogenic damage to the vital pulp–dentin complex underneath [2]. This protocol provides cost-effectiveness and desirable clinical outcomes [3].

Pulp protection or base/liner materials can be applied over the remaining demineralized dentin to promote the repair of carious dentin [4]. A clinical trial demonstrated that the use of liners was not directly associated with treatment success at 12 months [5]. However, clinicians who perform selective caries removal techniques still prefer to apply pulp protection materials over the remaining carious dentin [6,7]. It is expected that pulp protection materials will exhibit sufficient strength to ensure that the materials can withstand occlusal forces [8]. The materials should also promote the release of ions such as fluoride to encourage the remineralization of the demineralized dentin [9]. Additionally, the materials should not be highly toxic to pulpal cells [10].

The most commonly used pulp protection materials are resin-modified glass ionomer cements (RMGICs). RMGICs exhibit desirable clinical characteristics such as fluoride release, command set, and self-adhesion to dentin [11]. The main components of RMGICs are polyacids, fluoro–alumino–silicate glass, and water. Additionally, a photoinitiator and methacrylate monomers such as 2-hydroxyethyl methacrylate (HEMA) are added to the materials to enable free radical polymerization. HEMA also acts as the adhesion-promoting monomer that enhances the material’s penetration into conditioned dentin [12], thereby increasing the bonding strength of RMGICs to dentin. The hydrophilic functional group (-OH) in HEMA may encourage water sorption into the polymer network. This may lead to polymer plasticization, reducing the bulk mechanical strength of the materials [13]. However, the absorbed water may enable hygroscopic expansion, which is expected to balance the polymerization shrinkage [14]. This beneficial effect may help relieve the polymerization shrinkage stress of the materials [15]. Furthermore, the increase in water sorption may facilitate the acid–base reaction between the fluoro-alumino silicate glass and the polyacid, which could potentially increase ion release and promote dentin remineralization.

Various studies have reported the cytotoxic effects of HEMA on dental pulp cells [16,17,18,19], which could negatively affect tooth vitality. It has been speculated that incompletely polymerized RMGICs may release a substantial amount of unreacted HEMA monomers. These monomers may diffuse readily through the dentin barrier into the dentin–pulp complex due to their low molecular weight (130.14 g/mol) and hydrophilicity [20]. Furthermore, poor waste management in dental clinics may increase the risk of monomer contact with dental health care personnel or the contamination of the environment. Previous studies have therefore developed the liquid phase for RMGICs containing polyacids that were functionalized with methacrylate groups to enable light-curing mechanisms without the need for HEMA. A recent study prepared experimental RMGICs by mixing spherical pre-reacted glass fillers (SPG) and methacrylate-functionalized polyacids with added HEMA at 0 or 5 wt% [21]. The use of pre-reacted fillers was expected to enhance cross-linking between the polymer and the glass fillers, enhancing the mechanical properties [22,23]. The experimental materials exhibited comparable shear bond strength and surface microhardness with the commercial pulp protection material. The results from the previous study also suggest that the effect of adding HEMA at a low level (5 wt%) on the tested properties of the experimental RMGICs was negligible. 

The aim of the current study was to prepare the previously reported RMGICs using polyacid functionalized with methacrylate groups with 0 or 5 wt% of added HEMA. The powder components of the experimental RMGICs were spherical pre-reacted glass fillers. The degree of monomer conversion, gravimetric properties (mass/volume gain), biaxial flexural strength, ion release, and cytotoxicity of the materials were examined and compared with commercial materials. The research hypothesis was that the use of different liquid formulations would have no significant effects on the physical/mechanical properties and cytocompatibility of the experimental RMGICs. The second hypothesis was that experimental RMGICs would show comparable tested properties compared with the commercial materials.

## 2. Materials and Methods

### 2.1. Material Preparation

**Figure 1:**
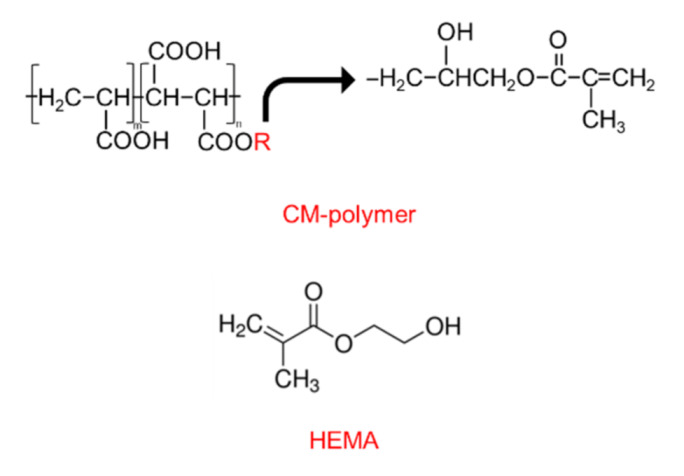
Chemical structure of the CM polymer and 2-hydroxyethyl methacrylate (HEMA).

Polyacids functionalized with methacrylate groups were prepared according to the protocol used in previous studies [21,24]. Briefly, the co-polymer of acrylic acid (Acros Organics, Fair Lawn, NJ, USA) and maleic acid (Sigma-Aldrich, St. Louis, MO, USA) was prepared using potassium persulphate (Honeywell Fluka, Charlotte, NC, USA) and isopropanol (RCI Labscan Limited, Bangkok, Thailand) as the initiator and chain transfer agent, respectively. Then, the co-polymer underwent a methacrylation process by reacting with glycidyl methacrylate (Sigma-Aldrich, St. Louis, MO, USA ) in tetrahydrofuran (RCI Labscan Limited, Bangkok, Thailand) under a nitrogen atmosphere. The catalyst and inhibitor used in the process were pyridine (RCI Labscan Limited, Bangkok, Thailand) and butylated hydroxytoluene, respectively (Honeywell Fluka, Charlotte, NC, USA). The final CM polymer (Figure 1) was then precipitated in diethyl ether and dried in a vacuum oven at room temperature. The molar mass assessed by gel permeation chromatography (Water 600E, Waters Corp., Milford, MA, USA) of the CM polymer was approximately 55,000 Dalton.

**Figure 2:**
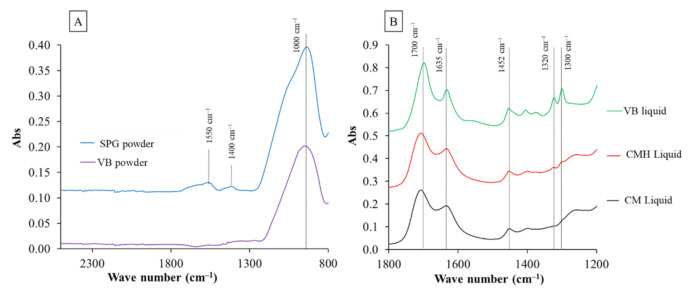
(**A**) The FTIR results of the powder phase; and (**B**) the FTIR spectra were obtained from the liquid phases of the RMGICs used in the current studies. VB is the commercial RMGIC (Vitrebond). Reproduced with permission under a Creative Common Attribution License from Thepveera et al. (2021). Reprinted with permission from [21].

The current study prepared two formulations of the liquid phase containing 0 or 5 wt% of 2-hydroxyethyl methacrylate (HEMA, Sigma-Aldrich, St. Louis, MO, USA) (Figure 1, Table 1). The liquids were added to water, camphorquinone (CQ, Sigma-Aldrich, St. Louis, MO, USA), and *N*,*N*′-dimethylaminoethyl methacrylate (DMAEMA, Sigma-Aldrich, St. Louis, MO, USA). The FTIR spectra (Nicolet iS5, Thermo Fisher Scientific, Waltham, MA, USA) of the CM and CMH liquids showed peaks representing the methacrylate group (C–O stretch, 1300 and 1320 cm^−1^) (Figure 2B).

**Figure 3:**
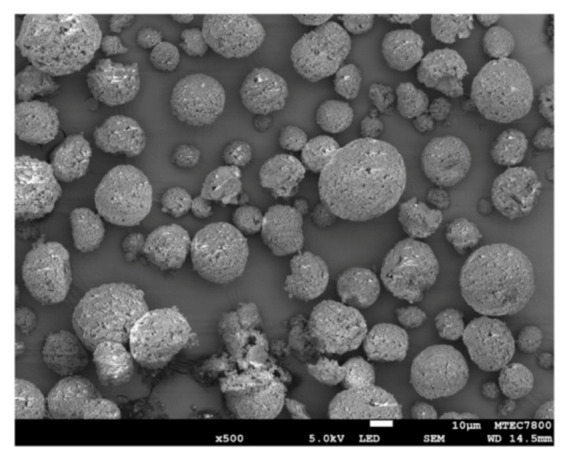
SEM image of spherical pre-reacted glass fillers (SPG) after the spray drying process. Reproduced with permission under a Creative Common Attribution License from Panpisut et al. (2020). Reprinted with permission from [25].

The powder phase of the experimental RMGICs comprised of spherical pre-reacted glass fillers (SPG, Figure 3). The fillers were prepared using the protocol described in previous studies [25,26]. Firstly, the fluoro–alumino–silicate glass (SiO_2_–Al_2_O_3_–CaF_2_–ZrO_2_) was prepared and mixed with 2 wt% of the CM liquid. The slurry mixture was prepared and sprayed to produce spherical fillers with a diameter of ~25 µm. The spherical glass fillers were mixed with the glass without spraying (irregular shape, mean diameter of 5 µm) using the mass percentage of 60:40 to maximize filler packing. The pre-reaction of acid–base neutralization producing polyacrylate salts (symmetric COO-stretch, 1470–1400 cm^−1^; asymmetric COO-stretch, 1600–1500 cm^−1^) in the SPG fillers was confirmed by the FTIR spectra of the powder phase (Figure 2).

The experimental RMGICs were formulated by mixing the SPG filler with the liquid phases of CM (F1) or CMH (F2) (Table 1). The powder-to-liquid ratio was fixed at 1.5:1 (mass ratio). The materials were then mixed using a plastic spatula on a mixing pad within 20 s. Commonly used resin-based pulp protection materials, including RMGIC (Vitrebond, lot no. BN981834, 3M ESPE, St. Paul, MN, USA) and Ca–Si-modified RMGICs (TheraCal LC, lot no. 1900006662, Bisco Inc., Schaumburg, IL, USA), were used as commercial comparisons. The commercial materials were prepared following the manufacturer’s instructions (Table 1).

### 2.2. Degree of Monomer Conversion

The degree of monomer conversion was assessed using FTIR-ATR (Nicolet iS5, Thermo Fisher Scientific, Waltham, MA, USA) (*n* = 3). Materials were prepared and placed in the circlip (10 mm in diameter and 1 mm in thickness; Springmaster Ltd., Redditch, UK) on the ATR diamond. The materials were then covered with an acetate sheet on the top surface. The materials were light-cured using an LED light-curing unit (irradiance of 1200 mW/cm^2^, SmartLite Focus Pen Style, DENTSPLY Sirona, York, PA, USA) for 40 s. The curing distance was set at ~3 mm from the top surface using a depth-of-cure testing guide (DENTSPLY Sirona, York, PA, USA) to mimic the use of the liner in the deep carious cavity. The test was performed at 25 ± 1 °C. FTIR spectra for the region from 800 to 2000 cm^−1^ before and after curing were recorded. The degree of monomer conversion ($D_{c}$, %) was calculated using the following equation [27]:

(1) $$D_{c}=\frac{100\left(\Delta A_{0}-\Delta A_{t}\right)}{\Delta A_{0}}$$

where $\Delta A_{0}$ and $\Delta A_{t}$ are the absorbance of the C–O peak (1320 cm^−1^) [28] above the background level at 1335 cm^−1^ before curing and after curing at time t, respectively.

### 2.3. Biaxial Flexural Strength (BFS) and Biaxial Flexural Modulus (BFM)

The materials were prepared and placed in a metal circlip (10 mm in diameter and 1 mm in thickness) to produce disc specimens (*n* = 5). The specimens were covered with acetate sheets and glass slides. They were then light-cured using the LED light-curing unit (SmartLite Focus Pen Style, DENTSPLY Sirona, York, PA, USA) for 40 s on the top and bottom surfaces [29,30,31,32]. They were left at room temperature for 24 h to allow polymerization to complete. The specimens were then removed, trimmed, and placed in deionized water (5 mL). The tubes were kept at 37 °C for 24 h prior to the test. Biaxial flexural strength (BFS) testing was performed using a ball-on-ring testing jig under the universal testing machine (AGS-X, Shimadzu, Kyoto, Japan). The test was conducted using a 500 N load cell with a crosshead speed of 1 mm/min. The load was applied until the materials fractured. BFS (Pa) was then calculated using the following equation [31]:

(2) $$\mathrm{BFS}=\frac{F}{d^{2}}\left\{\left(1+v\right)\left[0.485\ln\left(\frac{r}{d}\right)+0.52\right]+0.48\right\}$$

where F is the load at failure (N), d is the specimen’s thickness (m), r is the radius of circular support (mm), and v is Poisson’s ratio (0.3). Additionally, the biaxial flexural modulus (BFM, Pa) was calculated using the following equation:

(3) $$\mathrm{BFM}=\left(\frac{\Delta H}{{\Delta W}_{c}}\right)\times \left(\frac{\beta_{c}d^{2}}{q^{3}}\right)$$

where $\frac{\Delta H}{{\Delta W}_{c}}$ is the rate of change of the load with regard to the central deflection or gradient of force versus the displacement curve (N/m), $\beta_{c}$ is the center deflection junction (0.5024), and q is the ratio of the support radius to the radius of the disc.

Additionally, a representative fractured specimen of each group was selected and sputter-coated with Au at a current of 23 mA for 45 s by a sputter-coating machine (Q150R, Quorum Technologies, East Sussex, UK). The fracture surface was then examined under a scanning electron microscope with an accelerated voltage of 5 kV (SEM, JSM 7800F, JOEL, Tokyo, Japan).

### 2.4. Dimensional Stability (Mass and Volume Changes)

Disc specimens (10 mm in diameter and 1 mm in thickness) were prepared (*n* = 3). They were immersed in a tube containing 5 mL of deionized water. The tubes were incubated in an incubator at a controlled temperature of 37 °C for up to 8 weeks. At each time point (1, 4 h, 1, 2, 3, 4, 5 days, and 1, 2, 3, 4, 5, 6, 7, and 8 weeks), the specimens were removed and blotted dry. The mass and volume of the specimens were measured using a four-figure balance equipped with a density kit (MS-DNY-43, METTLER TOLEDO, Columbus, OH, USA). The specimen was then placed in a new tube with fresh deionized water. The percentages of mass (ΔM, wt%) and volume gains (ΔV, vol%) upon immersion in water were obtained using the following equations [31]:

(4) $$\Delta M=\frac{100\left[M_{t}-M_{0}\right]}{M_{0}}$$

(5) $$\Delta V=\frac{100\left[V_{t}-V_{0}\right]}{V_{0}}$$

where $M_{t}$ and $V_{t}$ are the mass and volume recorded at time t, respectively; $M_{0}$ and $V_{0}$ are the initial mass and volume, respectively.

### 2.5. Ion. Release

The storage solution (*n* = 3) from the mass and volume change studies was collected for the assessment of the ion release. 

#### 2.5.1. Fluoride Release

The storage solution was mixed with a TISAB III solution (Orion Ionplus, Thermo Fisher Scientific, Waltham, MA, USA) using a volume ratio of 1:10. Fluoride calibration standards at 0.1, 1, 10, and 100 ppm were prepared using a standard fluoride solution. The concentration of fluoride ions (ppm) in the mixed solution was assessed using a fluoride-specific electrode (Orion Versastar Pro, Thermo Fisher Scientific, Waltham, MA, USA). The cumulative level of fluoride release was calculated using the following equation:

(6) $$F_{c}=\sum_{0}^{t}F_{t}$$

where $F_{c}$ and $F_{t}$ are the cumulative fluoride ion concentration and fluoride concentration at time t, respectively.

#### 2.5.2. Aluminum, Calcium, Strontium, and Sodium Ion Release

The storage solution (*n* = 3) was mixed with 3 vol% nitric acid. The concentration (in ppm) of Al, Ca, Sr, and Na ions in the mixed solution was analyzed using inductively coupled plasma atomic emission spectroscopy (ICP-OES, Optima 8300, PerkinElmer, Waltham, MA, USA). The result was analyzed using Syngistix TM for ICP software v.2.0 (PerkinElmer, Waltham, MA, USA).

### 2.6. Cytotoxicity Test

Disc specimens were prepared and sterilized using UV light for 30 min on the top and bottom sides. Human dental pulp stem cells (hDPSCs) were purchased from Lonza (Lonza Group AG, Basel, Switzerland), so ethical approval was not required. The cells were maintained in a dental pulp stem cell basal medium (DPSCBM, Lonza Group AG, Basel, Switzerland) containing 10% of a dental pulp stem cell growth supplement (DPSCGS, Lonza Group AG, Basel, Switzerland) at 37 °C and a 5% CO_2_ humidified atmosphere. The cytotoxic effects on hDPSCs were measured using the methods described below.

#### 2.6.1. Direct Contact Cytotoxicity Test

Disc specimens (6 mm in diameter and 0.6 mm in thickness) were prepared (*n* = 5) and placed into a 96-well plate containing 200 µL of fresh DPSCBM. A standard tissue culture plastic was used as a control. The hDPSCs at Passage 4 were seeded at a density of 5 × 10^3^ cells/well. The cells were cultured for 3 days at a controlled temperature of 37 °C and a 5% CO_2_ humidified atmosphere. After 3 days of culture, an MTT viability assay was performed. DPSCs were incubated with a 0.2% 3-(4,5 dimethylthiazol-2-yl)-2,5-diphenyltetrazolium bromide (MTT) solution (Sigma-Aldrich, St. Louis, MO, USA) at 37 °C for 4 h. The reaction was stopped with 200 µL of dimethylsulfoxide (Sigma-Aldrich, St. Louis, MO, USA) and 25 µL of a glycine buffer (Research Organics, Cleveland, OH, USA). The end product’s color at an absorbance of 620 nm was measured using a spectrophotometer (Sunrise Absorbance Microplate Reader, Tecan Group Ltd., Männedorf, Switzerland). The results were reported as the relative cell viability (%) compared with the control using the following equation [33]:

(7) $$\mathrm{Relative}\;\mathrm{cell}\;\mathrm{viability}=\frac{\mathrm{OD}\;\mathrm{of}\;\mathrm{the}\;\mathrm{test}\;\mathrm{group}}{\mathrm{OD}\;\mathrm{of}\;\mathrm{the}\;\mathrm{control}}\times 100$$

where OD is the optical density.

#### 2.6.2. Indirect Contact Cytotoxicity Test

Disc specimens (*n* = 5) were prepared and immersed in 200 µL of DPSCBM and kept at room temperature for 5 h, then the discs were removed. The conditioned DPSCBM (extracts) was pipetted for 100 µL and mixed with 100 µL of fresh DPSCBM (diluted two-fold) and placed in the 96-well plate. The hDPSCs at Passage 4 were then seeded at a density of 5 × 10^3^ cells/well. The plain culture medium was used as the negative control. The cells were cultured for 3 days at a controlled temperature of 37 °C and a 5% CO_2_ humidified atmosphere. The MTT test was performed as described in Section 2.6.1.

### 2.7. Statistical Analysis

The results are shown in the current study as means ± SD. Prism 9.2 (GraphPad Software LLC., San Diego, CA, USA) was used to analyze the results. The Shapiro–Wilk test was used to analyze the normality of the data. Data were analyzed using one-way ANOVA followed by Tukey’s multiple comparisons. The repeated measured ANOVA and Tukey’s post hoc multiple comparisons were additionally used to assess the changes in mass and the volume gains of each material upon immersion in water for 8 weeks. All *p*-values below 0.05 were considered statistically significant. Sample size estimation was calculated using G*Power 3.1.9.6 (University of Düsseldorf, Düsseldorf, Germany) [34]. The effect sizes (Cohen’s f) were calculated using the results obtained from previously published studies [30,31]. G*Power indicated that the sample size used in each test exhibited a power of >0.95 in a one-way ANOVA (α = 0.05).

## 3. Results

### 3.1. Degree of Monomer Conversion

**Figure 4:**
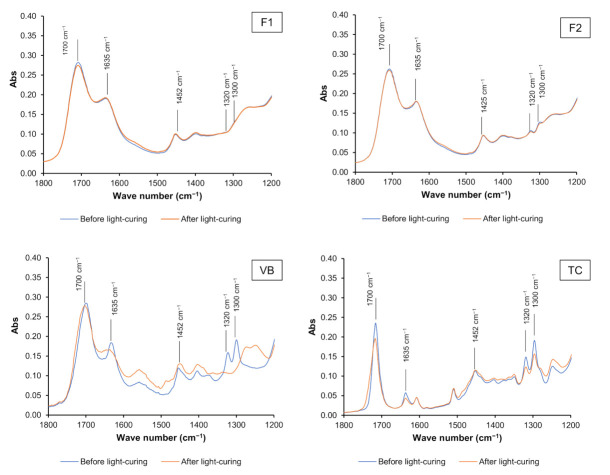
The FTIR spectra before and after the light-curing of the representative specimens in each group.

**Figure 5:**
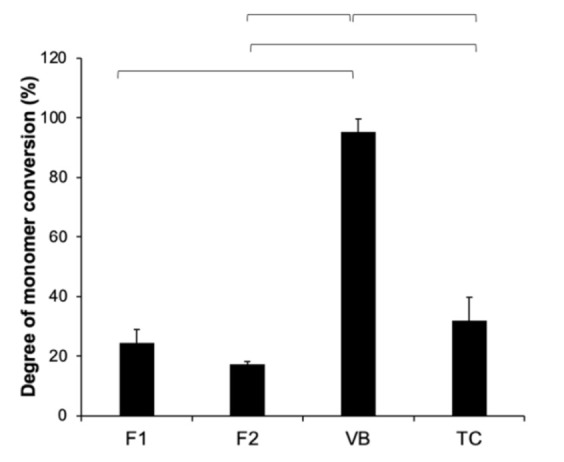
Degree of monomer conversion of all materials after light-curing for 40 s. Error bars are SD (*n* = 3). The lines indicate *p* < 0.05.

The reduction in the peak at 1320 cm^−1^ (C–O stretch, methacrylate) upon light-curing observed in F1 and F2 was less than that in VB and TC (Figure 4). The conversion of F2 (17 ± 1%) was comparable with that of F1 (25 ± 5%) (*p* = 0.3682). The conversion of VB (95 ± 4%) was significantly higher than that of F1, F2, and TC (34 ± 8%) (*p* < 0.05) (Figure 5). Additionally, the conversion of TC was significantly higher than that of F2 (*p* = 0.0312).

### 3.2. Dimensional Stability (Mass and Volume Changes)

**Figure 6:**
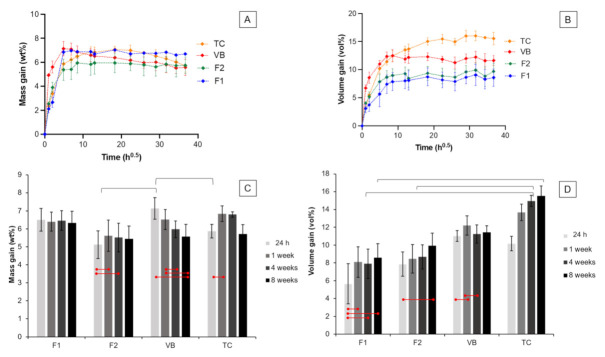
Plots of (**A**) mass gain and (**B**) volume gain versus the square root of time (h) for all materials after immersion in deionized water for up to 8 weeks. The (**C**) mass and (**D**) volume gains at 24 h, 1 week, 4 weeks, and 8 weeks. Error bars are SD (*n* = 3). Lines indicate *p* < 0.05.

At early time points, the mass and volume gains of each material increased linearly with the square root of time (h) (Figure 6A,B). The values then reached the maximum at 24–48 h, followed by a plateau phase for 8 weeks. The mass changes of F1 at 24 h (6.5 ± 0.6 wt%), 1 week (6.4 ± 0.5 wt%), 4 weeks (5.7 ± 2.2 wt%), and 8 weeks (6.3 ± 0.6 wt%) remained unchanged (*p* > 0.05) (Figure 6C). The mass gains of F2 at 1 week (5.6 ± 0.9 wt%) and 4 weeks (5.5 ± 0.8 wt%) were significantly higher than that obtained at 24 h (5.1 ± 0.8 wt%) (*p* < 0.05). The mass gain of VB at 24 h (7.1 ± 0.6 wt%) was significantly higher than that detected at 8 weeks (5.6 ± 0.7 wt%) (*p* = 0.0179). For TC, the mass gain at 24 h (5.9 ± 0.4 wt%) was significantly lower than that obtained at 1 week (6.8 ± 0.4 wt%) (*p* = 0.0199). At 24 h, the mass gain of VB was significantly higher than that of F2 (*p* = 0.0101) and TC (*p* = 0.0449). The mass gains of all materials at 1 week, 4 weeks, and 8 weeks were comparable (*p* > 0.05).

The volume gain of each group increased linearly and reached the maximum at approximately 1–2 weeks (Figure 6B). After that, the volume gains leveled off. The volume gain of F1 at 24 h (5.7 ± 2.2 vol%) was lower than that measured at 1 week (8.1 ± 1.7 vol%), 4 weeks (7.9 ± 1.6 vol%), and 8 weeks (8.6 ± 1.5 vol%) (*p* < 0.01) (Figure 6D). The volume gain of F2 at 24 h (7.9 ± 1.4 vol%) was significantly lower than that detected at 8 weeks (9.9 ± 1.4 vol%) (*p* = 0.0183). The volume gain of VB at 1 week (12.2 ± 1.1 vol%) was significantly higher than that at 24 h (11.0 ± 0.6 vol%) (*p* = 0.0091) and 4 weeks (11.2 ± 1.0 vol%) (*p* = 0.0244). The volume change of TC increased from 10.2 ± 0.8 vol% at 24 h to 15.5 ± 1.1 vol% at 8 weeks.

At 24 h and 1 week, the volume gains observed in all groups were comparable (*p* > 0.05). At 4 weeks, the volume gain of TC (15.0 ± 0.6 vol%) was significantly higher than that of F1 (*p* = 0.027) and F2 (*p* = 0.0334). At 8 weeks, the volume gain of TC was significantly higher than that of F1 (*p* = 0.0110).

### 3.3. Biaxial Flexural Strength (BFS) and Biaxial Flexural Modulus (BFM)

**Figure 7:**
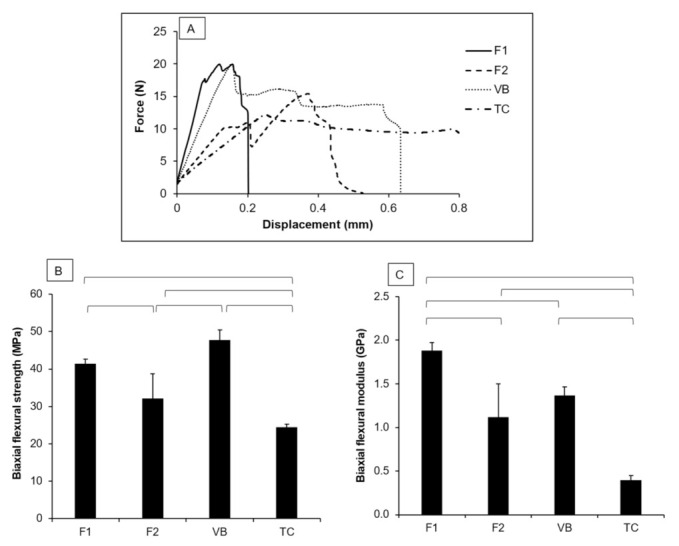
(**A**) Force–displacement of the representative specimens of each group. (**B**) Biaxial flexural strength and (**C**) modulus of the materials after immersion in water for 24 h. Error bars are SD (*n* = 5). The lines indicate *p* < 0.05.

**Figure 8:**
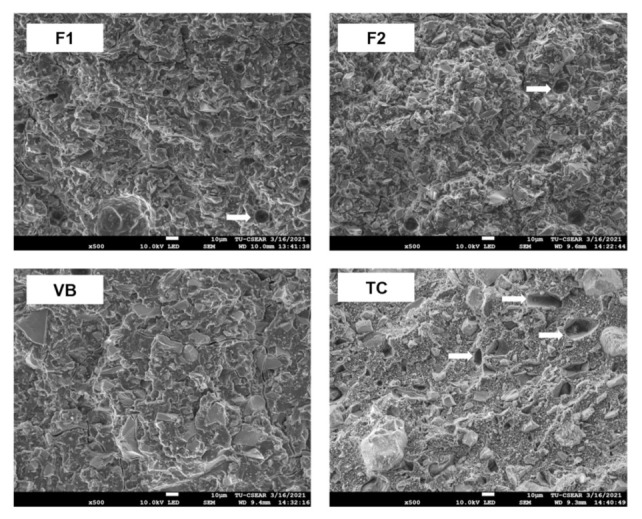
SEM images of fracture surfaces from a representative specimen of each group. Voids (arrows) are seen on the fracture sites of F1, F2, and TC.

The force–displacement diagram of F1 exhibited a rapid increase in force with minimal displacement compared with F2, VB, and TC (Figure 7A). The highest and lowest BFS values were obtained for VB (47.8 ± 2.6 MPa) and TC (24.4 ± 0.8 MPa) (Figure 7B). F1 (41.5 ± 1.2 MPa) showed a significantly higher BFS than both F2 (32.1 ± 6.6 MPa) and TC (*p* < 0.01). The BFS of F1 was comparable with that of VB (*p* = 0.0609). The BFM of F1 (1.9 ± 0.1 GPa) was significantly higher than that of F2 (1.1 ± 0.4 MPa), VB (1.4 ± 0.1 GPa), and TC (0.4 ± 0.1 GPa) (*p* < 0.01) (Figure 7C). F2 showed a BFM comparable with that of VB (*p* = 0.2554). Additionally, the BFM of TC was significantly lower than that of other materials (*p* < 0.01). The fracture surfaces of F1 and F2 showed a higher number of voids compared with VB (Figure 8). Multiple porosities were also detected with TC.

### 3.4. Ion. Release

**Figure 9:**
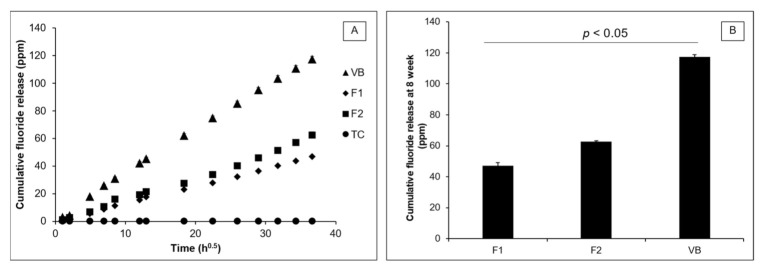
(**A**) Cumulative fluoride release versus immersion time (square root of time); and (**B**) cumulative fluoride release at 8 weeks from F1, F2, and VB. Error bars are SD (*n* = 3). The lines indicate *p* < 0.05.

The release of fluoride from F1, F2, and VB increased linearly with the immersion time (square root of time) (Figure 9A). No fluoride release was detected from TC. An early burst release of fluoride at 24 h was detected in VB (17.8 ± 0.5 ppm), F1 (5.5 ± 0.8 ppm), and F2 (6.8 ± 0.1 ppm). At 8 weeks (Figure 9B), the highest cumulative fluoride release was obtained from VB (117.4 ± 1.3 ppm), which was significantly higher than that of F2 (62.6 ± 0.6 ppm) and F1 (47.1 ± 2.1 ppm) (*p* < 0.01). The fluoride release of F2 was also significantly higher than that of F1 (*p* < 0.01). Sr, Ca, Al, and Na ions were detected with F1 and F2 (Table 2). For VB, the concentrations of Sr and Ca were lower than the detection limit. TC also released Ca, Sr, and Al. However, the level of Na in TC was lower than the detection limit.

### 3.5. Cytotoxicity Test

**Figure 10:**
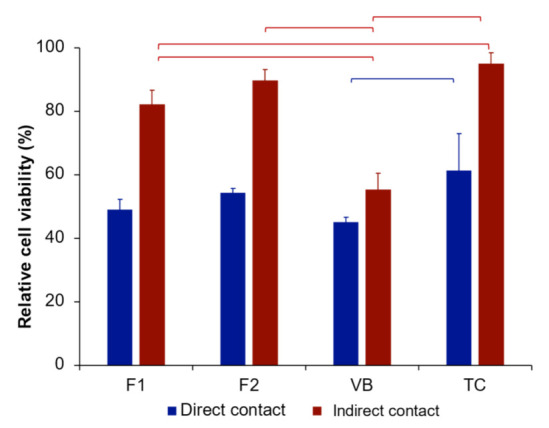
Relative cell viability of human dental pulp stem cells for each material from direct contact and releasing contact assessments. Error bars are SD (*n* = 5). The lines indicate *p* < 0.05.

For the direct contact cytotoxicity assessment, the highest and lowest relative cell viability values were obtained with TC (61 ± 12%) and VB (45 ± 1%), respectively (Figure 10). The relative cell viability of TC was significantly higher than that of VB (*p* < 0.01). Additionally, the relative cell viability of F1 (49 ± 3%) was comparable with that of F2 (54 ± 1%) and VB (*p* > 0.05).

For the indirect contact cytotoxicity assessment, the highest and lowest relative cell viability values were observed with TC (95 ± 3%) and VB (55 ± 5%). F1 (82 ± 5%) showed comparable relative cell viability with F2 (89 ± 3%) (*p* = 0.1596). TC showed comparable relative cell viability with F2 (*p* = 0.8964). The relative cell viability of F1, F2, and TC was significantly higher than that of VB (*p* < 0.01).

## 4. Discussion

A previous study successfully prepared resin-modified glass ionomer cements containing polyacid functionalized with methacrylate groups [21]. The use of different liquid phases containing HEMA (0 or 5 wt%) showed negligible effects on the rheological properties, surface microhardness, and shear bond strength to dentin. The use of HEMA at a low level (0–5 wt%) was expected to reduce concerns regarding the cytotoxic effects of the experimental RMGICs.

The current study additionally assessed the degree of monomer conversion, dimensional stability (mass/volume changes), biaxial flexural strength, ion release, and cytotoxicity to human dental pulp stem cells. The results showed that the use of different liquids (CM or CMH) affected the biaxial flexural strength of the experimental RMGICs. Hence, the first research hypothesis was rejected. Additionally, the second research hypothesis was also rejected, as the experimental materials showed significantly higher relative cell viability than VB.

### 4.1. Degree of Monomer Conversion

A high level of monomer conversion in resin-based dental materials is required to ensure that the materials have good physical and mechanical properties [35,36,37]. It should be mentioned that the minimum degree of monomer conversion has not yet been specified in the ISO standards. The degree of monomer conversion is governed by the chemical structure of monomers, light transmission/intensity, the degree of refractive index mismatch, and the thickness of the materials. The highest monomer conversion was observed in VB. In general, the use of monomers with low glass transition temperature usually results in the high conversion of monomers [38]. Hence, the use of HEMA (T*_g_*~−60 °C) [39] as the primary base methacrylate monomer could help promote the monomer conversion of the material. Additionally, the low viscosity of HEMA monomers may facilitate the mobility of reactive chains, which could increase the degree of monomer conversion [40]. The conversion of VB detected in the current study was higher than that reported in a previously published study (60–80%) [41]. This could be due to the effects of different light intensities from the curing unit. The light intensity of the light-curing unit used in the current study was approximately 1000–1300 mW/cm^2^, whilst that used in the previously published study was ~500 mW/cm^2^. The increase in light intensity helped enhance the formation of free radicals, which could have subsequently increased the polymerization of the materials [42].

The main reason for the low monomer conversion observed with the experimental RMGICs could have been due to the limited mobility of the high molecular weight polyacid. This may have led to the low reactivity of free radical chains, resulting in a low degree of monomer conversion. It was speculated that the addition of HEMA may help enhance the free radical polymerization of the materials. The results from the current study, however, indicated that the addition of HEMA had no significant effect on the degree of monomer conversion in the experimental RMGICs. This could have been because the level of HEMA was insufficient to significantly increase the polymerization of the material. Another possible reason could be that the level of CQ in the experimental RMGICs was not sufficient to generate free radicals for rapid polymerization. Hence, the light-cured setting mechanism of the experimental RMGICs needs to be improved to aid the command set for clinicians. Possible strategies to enhance the polymerization of the experimental RMGICS could be either to increase the level of CQ or to use alternative photoinitiations such as Lucirin-TPO, which is more efficient at absorbing light and converting photon energy compared to CQ [43].

The conversion of TC in the current study was higher than that reported in the published study (~10%) [44]. This could be due to the extended light-curing in the current study (40 s) compared with the protocol used in the published study (20 s). Additionally, the thickness of the specimen used in this study was 1 mm, whilst that in the published study was 4 mm. The greater thickness of highly opaque TC may have reduced the light transmission of the material [44]. This emphasizes the importance of the clinical practice of limiting the thickness of TC and extending the light-curing time beyond the manufacturer’s instructions to enhance the free radical polymerization of the material.

### 4.2. Dimensional Stability (Mass and Volume Changes)

RMGICs in cavities will be exposed to dentinal fluids which could lead to water sorption, mass gain, and hygroscopic expansion [45]. The expansion of resin-based materials was also expected to relieve polymerization shrinkage stress [14,46]. The previous study showed that the shrinkage stress of ion-releasing materials as reduced by 62–100% after water immersion [46]. This could potentially reduce the risk of debonding at the tooth-restoration interface caused by shrinkage stress.

The mass and volume gains of RMGICs in the current study were within the range of commercial RMGICs reported in a published study (mass gains of ~4–10 wt%, volume gains of ~5–15 vol%) [30]. The mass gain of VB was reduced with immersion time. The loss of mass may be the result of glass dissolution or the elution of components from the material [45]. It was speculated that HEMA polymers may not be sufficiently rigid because the monomers consist of a methacrylate group only at one end or because the hydroxyl group formed hydrogen bonds with water. The absorbed water could plasticize the HEMA polymer and expand the polymer network [13]. This may result in the hydrolytic degradation of methacrylate monomers and the release of components [13,47].

The mass gain of F1 at each time point was more stable than that of other materials, as was expected. This could be due to the more rigid matrix of F1 resulting from the lack of hydrophilic HEMA monomers. The low flexibility of the matrix may, however, reduce the release of ions [26]. The highest volume gain was detected for TC. The hydration of the Ca–Si cement may increase through water sorption, expanding the material. The water sorption increased the mass and volume of the materials, but this may negatively affect the strength of the materials [48].

### 4.3. Biaxial Flexural Strength

The high flexural strength of pulp protection material is essential to ensure that the materials can withstand the applied mechanical forces during functioning. The flexural strength of the experimental RMGICs was 32.1–41.5 MPa, which was higher than that (10 MPa) required by BS ISO 9917-2:2017 Dentistry—Water-based cements (Part 2: Resin-modified cements) [29]. This may suggest that the materials would pass the requirements of the standard. It should be mentioned that the flexural strength test indicated in the ISO 9917-2:2017 standard was a three-point bending test. The current study used the biaxial flexural strength test, which requires less material, and the specimens’ preparation was less sensitive than that of the three-point bending test. For example, disc specimens in the biaxial flexural test can be easily light-cured due to their similar diameter to the light-curing tip [49]. Furthermore, disc specimens may be less likely to fail due to edge failure compared with a beam specimen in the three-point bending test. Additionally, it has been reported that the results obtained from the biaxial flexural strength test were consistent with those of the three-point bending test but with minor variations [50].

The use of CMH liquid significantly reduced the BFS and BFM of the experimental RMGICs. This could be because the HEMA enhanced water sorption, plasticizing the polymer network and thus reducing the strength and modulus of the elasticity of the materials [51]. A previous study demonstrated that F1 and F2 showed higher surface microhardness than VB [21]. However, the biaxial flexural strength of VB was comparable with that of F1 but higher than that of F2. It can be speculated that the bulk mechanical strength of F1 and F2 may be reduced by the presence of voids, which may act as crack propagators, in the experimental RMGICs [52,53]. The voids may occur due to air entrapment during hand-mixing or the dissolution of glass.

The flexural strength of TC was comparable with that reported in a published study (~20–30 MPa) [54]. TC exhibited the lowest strength among the materials. This could be due to the sub-optimal polymerization of the materials [44]. Additionally, Ca–Si cements are highly hydrophilic, which may have led to an increase in the water sorption and solubility of TC [55]. This may have subsequently reduced the mechanical properties of the material. The flexural strength test in the current study was performed at 24 h. Future work should use a longer aging time to assess the long-term mechanical properties of the material.

### 4.4. Ion. Release

The most attractive property of glass ionomer cements is their ability to release fluoride. It is expected that the sustained release of fluoride at a low concentration could potentially help promote the remineralization of the demineralized tooth minerals [56]. However, the minimum level of fluoride release required to promote a significant clinical benefit has not yet been established by the ISO standards [57]. The fluoride release occurred in two main steps, including early burst release, followed by the slow and sustained diffusion-controlled release. The highest fluoride release was observed from VB, followed by F2 and F1. This trend may be related to the level of HEMA contained in the materials. It can be speculated that the water absorbed by HEMA may help enhance glass dissolution via acid–base reactions [46,51]. Additionally, the plasticizing effect of water may increase the flexibility of the polymer network, which could subsequently enhance the diffusion of fluoride [25].

Glass ionomer cements can release other ions in addition to fluoride. The experimental RMGICs released more Al, Sr, and Ca than VB. This may be due to the different glass networks between SPG and the powder phase of VB. The ion-releasing properties are believed to enhance the bioactivity of the materials at the tooth–restoration interface. The ions may induce biomimetic tooth repair by encouraging the intrafibrillar mineralization of demineralized collagen [58]. The release of calcium is expected to promote the precipitation of hydroxyapatite in demineralized collagens. Additionally, strontium could stabilize the precursors for mineral apatite precipitation [59]. This mineralization could potentially help protect collagen from further proteolytic degradation and increase the clinical longevity of the restored tooth. The co-presence of strontium and fluoride ions may produce a synergistic effect for anticaries actions [60]. The limitation of the test was that the measurement of Al, Sr, Ca, and Na ions using ICP-OES was performed at the final time point. Future studies should focus on measurements at various time points to determine the release kinetics. 

### 4.5. Cytotoxicity Test

Pulp protection materials (base/liner) are commonly used in deep cavities where the thickness between the pulp–dentin complex and the cavity is inadequate to protect the stimuli of vital cells in the pulp. Additionally, the diameter of the deep dentin increases with the increase in the depth of the cavity. This may increase the risk of toxic components leaching into the pulp. Low molecular weight monomers may readily diffuse through the dentin barrier, reaching dental pulp cells. HEMA causes cytotoxic effects by increasing reactive oxygen species, leading to oxidative DNA damage [61].

It was stated by the ISO 10993-5:2009 standard that materials may be considered to have cytotoxic potential if their obtained relative cell viability is lower than 70% compared with the blank control [62]. The relative cell viability obtained from the indirect contact cytotoxic test was higher than that obtained from the direct contact test, as was expected. The elution (extract) from the materials in the indirect contact test was diluted (1:1 volume ratio). This may have subsequently reduced the cytotoxic effects of the materials in the indirect contact test. However, the indirect toxicity (extract study) may be more clinically relevant than the direct contact test, as RMGICs are not supposed to be placed directly on the exposed pulp tissues.

TC showed the highest relative cell viability. This may support the high cytocompatibility of Ca–Si cements reported in various published studies [63,64,65]. The finding may additionally confirm the suitability of TC for direct application over the exposed pulp tissue (direct pulp capping) to promote mineralizing effects. It is believed that the primary component that caused the cytotoxic effects of RMGICs to hDPSCs was HEMA. The results from the indirect contact test demonstrated that only VB (55%) showed a relative cell viability lower than 70%, which was considered to show cytotoxic potential according to the BS EN ISO 10993-5: 2009 standard. VB contained HEMA at a higher concentration (20–30 wt%) compared with the experimental materials. The hydrophilic groups in the HEMA polymers may therefore encourage water sorption and enhance the hydrolytic degradation of the polymer [66,67].

F2 showed comparable relative cell viability with F1. This may be because the level of HEMA in F2 was too low to induce significant cytotoxic effects. The limitation of the current study was a lack of results regarding monomer release. An elution study should be performed in future work to determine the potential release of substances from the materials that are toxic to cells. The high level of relative cell viability obtained from the experimental RMGICs may be beneficial for maintaining pulpal repair capacity or promoting dentin formation. This may consequently increase the longevity of the restored tooth.

In terms of clinical applications, the experimental RMGICs exhibited acceptable rheological properties and shear bond strength to dentin comparable with the commercial RMGIC for pulp protection [21]. The strength of the materials at 24 h was also higher than that required by the ISO standard, which may suggest that the materials can withstand occlusal forces. The materials also released multiple ions that could potentially help remineralize the demineralized dentin [68]. Additionally, the toxicity determined by the indirect assessment of the experimental RMGICs was in the acceptable range according to the standard. However, the degree of monomer conversion after the light-curing of the experimental RMGICs was lower than that reported for resin-based materials (>50%) [69]. This may reduce the immediate strength of the materials, which could affect the restorative procedure. Future work will mainly have to focus on the optimization of the polymerizing efficacy of the experimental RMGICs. It should be emphasized that this study is an in vitro study. Hence, the clinical significance of the results should be carefully interpreted.

## 5. Conclusions

The experimental RMGICs containing methacrylate-functionalized polyacids and spherical pre-reacted glass fillers exhibited lower monomer conversion and fluoride release than the commercial RMGIC. The dimensional stability of the experimental materials was within the range of that observed with the commercial materials. The experimental RMGICs showed a higher level of cell viability in the indirect contact test than the commercial RMGIC. The use of liquid formulation containing 5 wt% HEMA reduced the mechanical strength of the experimental RMGICs but enhanced ion release. Additionally, the strength of the experimental materials was higher than that required by the standard.

## Figures and Tables

**Table 1:** Composition of materials used in the current study (pph means parts per hundred).

Materials	Composition	Instructions
Formulation 1 (F1)	Powder: spherical pre-reacted glass fillers (SPG) (40 wt% irregular shape, 60% spherical shape) Liquid: CMH liquid; CM polymer (55 wt%), water (45 wt%), tartaric acid (2 pph), CQ (0.7 pph), DMAEMA (1.4 pph)	- Weigh the powder and liquid components (PLR 1.5:1 mass ratio) - Hand-mix within 20 s on a mixing pad - Light-cure by an LED light-curing unit for 40 s
Formulation 2 (F2)	Powder: spherical pre-reacted glass fillers (SPG) (40 wt% irregular shape, 60% spherical shape) Liquid: CMH liquid; CM polymer (50 wt%), HEMA (5 wt%), water (45 wt%), tartaric acid (2 pph), CQ (0.7 pph), DMAEMA (1.4 pph)	
Vitrebond (VB)	Powder: glass powder (>95 wt%), diphenyliodonium chloride (<2 wt%) Liquid: copolymer of polyacids (35–45 wt%), HEMA (20–30 wt%), water (30–40 wt%)	- Dispense one scoop of powder and one drop of liquid (PLR 1.4:1 mass ratio) - Hand-mix within 10–15 s on a mixing pad - Light-cure by an LED light-curing unit for 30 s
TheraCal LC (TC)	Calcium silicate cement (30–50 wt%), polyethylene glycol dimethacrylate (10–30 wt%), barium zirconate powder (1–10 wt%)	- Inject material from the syringe - Light-cure by an LED light-curing unit for 40 s

**Table 2:** Cumulative aluminum, calcium, strontium, and sodium ion release at 8 weeks.

Materials/Ion (Mean and SD, ppm)	Sr	Ca	Al	Na
F1	4.12 (0.15)	1.85 (0.06)	0.40 (0.04)	0.77 (0.03)
F2	6.31 (0.09)	3.08 (0.05)	0.21 (0.01)	0.58 (0.03)
VB	<0.1 *	<0.1 *	0.26 (0.01)	15.02 (0.21)
TC	8.22 (0.19)	26.91 (0.98)	0.29 (0.01)	<0.01 *

*: The concentration was lower than the detection limit.

## Data Availability

The datasets generated and/or analyzed during the current study are available from the corresponding author upon reasonable request.
